# Body Balance of Children and Youths with Visual Impairment (Pilot Study)

**DOI:** 10.3390/ijerph191711095

**Published:** 2022-09-05

**Authors:** Katarzyna Walicka-Cupryś, Maciej Rachwał, Agnieszka Guzik, Paweł Piwoński

**Affiliations:** Department of Physiotherapy, Institute of Health Sciences, College of Medical Sciences, University of Rzeszów, 35-310 Rzeszów, Poland

**Keywords:** postural stability, blind, proprioceptive

## Abstract

Aim: The study was designed to assess the effects of surface instability in the response of the balance control system in children and youths with visual impairment (BL) and in normally sighted controls (NE). Materials and Methods: The empirical research study involved 80 individuals, aged from 6 to 20 years, with a mean age of 14.37 (±4.68), including 40 blind individuals and a randomly selected control group 40 normally sighted. Stabilometric measurements were performed with the use of the Platform CQ Stab 2P, with eyes open (EO) and closed (EC) on the solid surface, and then, the same procedure was performed on the platform covered with 1-centimetre-thick foam. Results: Statistical analyses (Wilcoxon matched-pairs test, Mann–Whitney U test) of the results identified during the trials reveal the following findings in the BL group in the EO and EC tests. The results of the foam surface test were higher and the differences were statistically significant in the BL group (sway path EO *p* = 0.009, EC *p* = 0.006; mean amplitude EC *p* = 0.030; mean velocity EO *p* = 0.009, EC *p* = 0.006; sway area EO *p* = 0.017, EC *p* = 0.009; and number of COP deflections along the sagittal plane EO *p* = 0.004). No similar correlations were observed in the NE group, except for the mean amplitude EO *p* = 0.033 and sway area EO *p* = 0.030. There was one difference between the BL and the NE group for the mean amplitude parameter, *p* = 0.018, in a solid surface test with open eyes. The results were higher in the BL group. Conclusions: The present study showed no worse balance in the BL group than in the NE group but worse performance on the foam than without it. It indicates the need to develop body balance skills in blind people by improving their proprioceptive sensitivity. In everyday life and training, blind people should experience exteroceptive stimuli, different textures, and unstable surfaces as much as possible.

## 1. Introduction

The organ of vision plays a role in maintaining balance, as shown by numerous studies [1,2,3,4,5,6,7]. Peterson et al. [8] established that up until 12 years of age, children cannot fully use vestibular and visual stimuli in the process of balance control. Furthermore, Steindl et al. [9] showed that the ability to fully use vestibular and visual stimuli in balance control developed at 15–16 years of age. Mraz et al. [10] reported increased posturographic parameters (an increase in the sizes of the stabilogram’s surfaces and its total length) in female subjects aged 8–22 years if visual control was not applied. The relative stability of the Centre of Pressure (COP) excursion is observed during the period of life between 15 and 65 years of age [9,11].

Sipko and Skolimowski [12] report that postural balance control mechanisms do not function normally in individuals with visual impairment, leading to such adverse effects as a postural imbalance. Compensatory mechanisms occurring in blind individuals make it possible to reduce the effects of the disability. However, research shows that other sensory functions are not more developed in people with visual impairments, but they use them more effectively. In addition, a lack of visual input at the stage of motor development results in abnormal motor coordination [12,13,14]. Paszko-Patej et al. [14] report that factors adversely affecting neuromuscular coordination, e.g., nervous system injury, mental disability, or postural defects, lead to impaired balance control; on the other hand, visual or hearing impairments result in greater focus and attention to activities performed, leading to more accurate maintenance of postural stability. Conversely, Houwen et al. [15] assessed motor performance in school-age children with visual impairments and reported that static and dynamic balance in these subjects was poorer than in healthy peers.

In individuals with visual impairments, the interaction of the somatosensory and vestibular systems may be augmented, as a result of which these functions play the main role in balance maintenance, and they compensate for insufficient visual input. Schwesig et al. [13] point out that when subjects with visual impairments rely more on visual stimuli, their capacity to maintain postural stability is lower than in a situation when the vestibular system is involved.

Literature review shows a scarcity of research reports discussing dynamic balance and the effects of proprioception in postural control in individuals with visual impairments [13,15]. This conclusion motivated the current study. The quantification of balance in the Romberg protocol, i.e., with eyes open and closed in blind individuals, was deliberately carried out. Our research and the literature indicate that there may be differences in the open-eyed and closed-eyed tests in blind individuals. It may indicate an increased involvement of proprioceptive stimulus analysis, following a command associated with increased caution. The measurement was conducted on a two-plate platform, and exteroceptive perception was impaired with foam. The foam was lightweight, moderate density (55 kg/m^3^), and 1 cm thick. The feet in both trials were positioned in free stride, allowing for repeatability of measurement.

The study was designed to assess the effects of surface instability in the response of the balance control system in blind children and youths compared to sighted controls. The hypothesis was that blind individuals would have worse balance characteristics.

## 2. Materials and Methods

### 2.1. Study Participants

After applying the inclusion and exclusion criterion, the analysis contained 80 people who did not meet the criteria for physical activity, according to the index classification of Moderate-to-Vigorous Physical Activity—MVPA. Participants came from the Subcarpathian region of Poland, were aged 6 to 20 years, with a mean age of 14.37 (±4.68) years, a mean body height of 151.52 cm (±19.99), and a body weight of 51.81 kg (±20.67). In this study, there were 40 participants in the blind group (BL) and 40 participants in the control group (individuals with normal eyesight (NE)), including 20 women and 20 men in each group, aged 6 to 20 years. The mean body height BL was 150.84 cm (±20.59) and NE 152.2 (±19.38), and the body weight BL was 51.40 kg (±21.13) and NE 22.20 kg (±20.21). According to the WHO classification, the study population is children and youths [16,17,18].

### 2.2. Study Qualification

**Figure 1:**
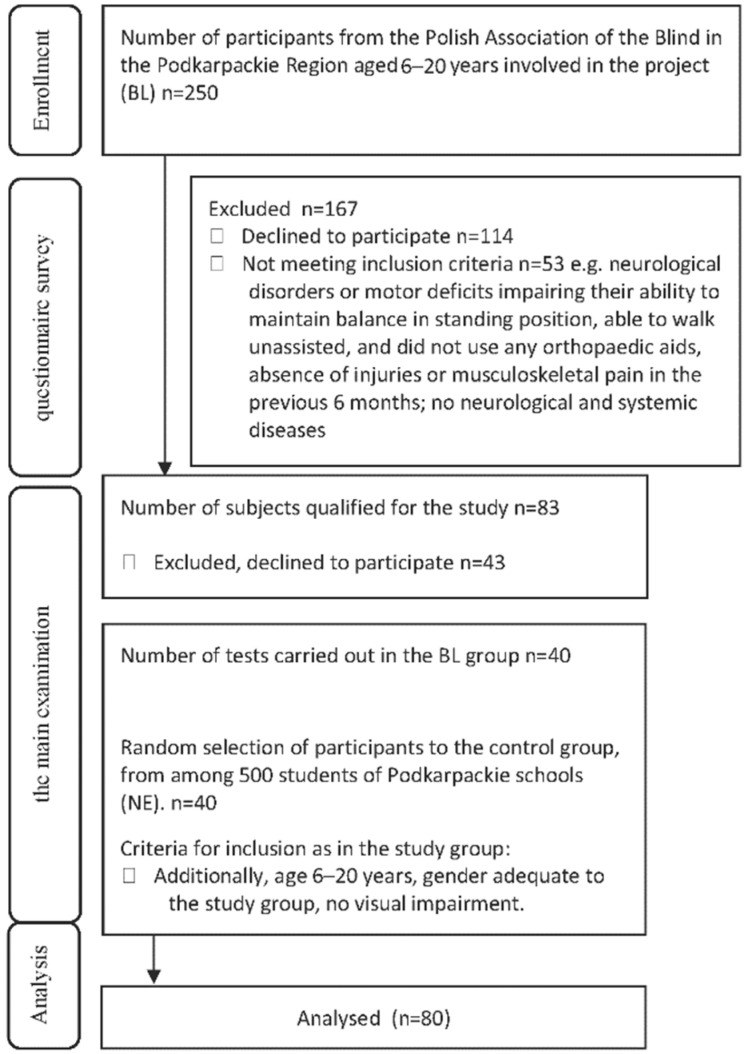
Flow Diagram.

The study was divided into three phases. In the first stage, declarations related to participation in the study were submitted by the parents of the children and the adult persons, aged 6–20, who were members of the Polish Association of the Blind’s Podkarpackie Region Branch (250 subjects). In the second stage, the parents or adult participants completed questionnaires, based on which, subjects qualified for the next stage (83 individuals). During the third stage, the subjects were examined for the essential anthropometric characteristics, body height and mass, and took part in a balance test (40 individuals). The statistical power of our study was 0.80, the same as the recommended power of 0.8, and the required number of people per group was 40. The eligibility criteria are shown in Figure 1.

Ultimately, all the study stages were completed by 40 blind individuals (BL)—who had certificates of congenital visual impairment ICD 10—H54 and blindness was defined as visual acuity of less than 3/60 (0.05) or corresponding visual field loss in the better eye with best possible correction [19,20]. All the individuals were in the normal development of intellectual capacities and did not meet the criteria for physical activity, according to the index classification of Moderate-to-Vigorous Physical Activity—MVPA. (Physically inactive people are not physically active for 7 days a week, adding up all physical activities during the day for one hour a day) [21,22].

Physically active people are physically active for 7 days per week, with cumulative physical activity for a minimum of 1 h per day. Physical activity includes all activities that accelerate breathing and heart rate, e.g., cycling, sports game, and physical education at school.

After eligibility criteria were met, the control group (individuals with normal eyesight and no need for correction (NE), matched for age and gender to the study group) was randomly selected from among 500 students, inactive by the index MVPA classification, and attending schools in the Podkarpackie Region, Poland. All the persons recruited for the study could walk unassisted and did not use any orthopaedic aids (canes, crutches, or walkers). All the subjects were able to assume the standing position for the assessment. Individuals with neurological disorders or motor deficits, impairing their ability to maintain balance in a standing position without aid, and/or those using any orthopaedic tools were excluded. The eligibility criteria were as follows: absence of injuries or musculoskeletal pain in the previous six months, because it has been proven in experimental studies that muscle tissue is completely regenerated after about 3 to 6 months [23,24]; and no neurological and systemic diseases. The information regarding health status and physical activity from the index MVPA, orthopaedic aids, and pain was obtained before the examinations were carried out, along with consent for participation.

The local bioethics commission approved the study.

### 2.3. Methods

#### 2.3.1. Anthropometric Measurements

All the measurements were performed on the same day, starting with anthropometric measurements. Body height was measured with a Seca 213 mobile stadiometer, with an accuracy of 0.1 cm. Body mass was measured using the electronic scale, OMRON BF 500, with an accuracy of 0.1 kg. The measurements were performed under standard conditions; children were in underwear and barefoot, stood upright, without bending knees.

#### 2.3.2. Balance Test

The balance parameters were recorded using the Stabilometry Platform CQ Stab 2P (CQ Elektronik System) [25,26,27,28,29]. The subject was asked to stand with legs straight and naturally spread to match the width of their hips; the gaze was fixed at a point located at the level of their eyes, at a distance of two meters [26,30]. Before the trial, with eyes open, each subject was instructed to look at a specific point on the wall and to refrain from moving their arms and head. Blind individuals were asked to turn their heads straight ahead. The examination consisted of four 30 s-long trials held in succession. The first trial involved assessment in a relaxed standing position, with eyes open (EO); the second trial was performed with no visual control of the spatial arrangement of the body, i.e., with eyes closed (EC) [31]. Blind individuals also include those with a sense of light and the imagery of closing the eyes can have an internal biofeedback effect, and it has been assumed that it can also affect balance parameters. Our observations and the literature confirm this. In many studies of blind people, closed-eye tests are also performed. The command has cognitive significance [32,33]. During EC trials, the examiner ensured that the subject could maintain balance without visual input. The measurement started 10 s after the subject reported they were ready. During the second stage, the trials with eyes open and closed followed the same procedure but were performed on the platform covered with 1-centimetre-thick foam [13,34], applied as proprioceptive interference. During the second measurement preparation, the participant rested in a standing position in a designated area in front of the platform. The time between examination without and with the foam was about 30 s. During this period, the preparation of the workstation and calibration continued. The examination was performed in a soundproofed room; the subjects were barefoot during the measurement. Based on COP excursions, the following rates were calculated:

- Sway path (SP) (mm)—the total length of the path in the sagittal and frontal plane;
- Mean amplitude (MA) (mm)—mean deflection of the COP;
- Mean velocity (MV) (mm/s)—mean speed of COP excursions along the sagittal and frontal plane;
- Sway area (SA) (mm^2^)—area delineated by the COP;
- LWAP—number of COP deflections along the sagittal plane;
- LWML—number of COP deflections along the frontal plane.

### 2.4. Statistical Analysis

The acquired material was subjected to statistical analyses, computed using Statistica 12.5. The normality of distributions was verified with the Shapiro–Wilk W test, and equality of variances was assessed with Levene’s test. A nonparametric Mann–Whitney U test for independent samples was used to assess the differences in the mean values of the balance parameters between the blind subjects and the controls. Intragroup variability for the same subjects was examined using a Wilcoxon matched-pairs test in two measurements on the solid surface and the foam surface. Statistical significance was assumed at *p* < 0.05.

## 3. Results

Statistical analyses of the results identified during the trials on a foam surface and a solid surface, with eyes open—EO, in the BL group, performed using a Wilcoxon matched-pairs test, showed several significant differences in the following parameters: sway path SP-EO (*p* = 0.009), mean velocity MV-EO (*p* = 0,009), sway area SA-EO (*p* = 0.017), and number of COP deflections along the sagittal plane LWAP-EO (*p* = 0.004) (Table 1). Higher results were found in the measurements on the foam surface in all of the above parameters. A comparison of the results in the BL group, in the trials with eyes closed—EC, on a foam surface and a solid surface, showed statistically significant differences in the sway path SP-EC (*p* = 0.006), mean amplitude MA-EC (*p* = 0.030), mean velocity MV-EC (*p* = 0.006), and sway area SA-EC (*p* = 0.009) (Table 1). Higher results were found in the measurements on the foam surface in all of the above parameters.

In the NE group, the trials with eyes open, on the foam surface and solid surface, produced statistically significant results in the following parameters: mean amplitude MA-EO (*p* = 0.033), and SA-EO (*p* = 0.030) (Table 2). The results in all the above parameters were higher in the measurements on the foam surface. The trials with eyes closed on foam and solid surfaces in the NE group showed no statistically significant differences in the results. The results for MA-EC (*p* = 0.052), SA-EC (*p* = 0.052), and MA-EC (*p* = 0.067) were approaching the threshold of statistical significance (Table 2).

Statistical analyses using the Mann–Whitney U test showed no significant differences between the results acquired by subjects in the BL and the NE groups, in trials with eyes open on the foam surface (Table 3). Similarly, the trials with eyes closed on the foam surface produced no significant differences between the study group and the controls (Table 3).

In the trials with eyes open, on a solid surface, the findings show statistically significant differences between the results of the BL and NE groups in MA-EO (*p* = 0.018) (Table 4). No significant differences were observed in the trials with eyes closed on a solid surface (Table 4).

## 4. Discussion

This study investigated the effects of surface instability on the response of the balance control system in people with visual impairment (BL) and normally sighted controls (NE). The significant findings of the present study did not show poorer balance in the BL group compared to the NE group. However, in the tests on the unstable surface, considerable sensitivity to the interfering factor’s effects was observed in the BL group. Therefore, the results do not support the hypothesis that blind children and youths have poorer balance characteristics than sighted peers.

We took up this subject matter in our study because balance-related problems resulting from proprioceptive disturbances (e.g., induced by an unstable foam surface) in people with visual impairments and normally sighted controls are under-researched [13,15]. Furthermore, it appears that COP excursions are only affected to a small degree by eyesight, shown in a population aged 12–15 years [35]. It was reported that balance parameters did not differ significantly in trials with eyes open and closed. Hence, it is likely that the balance control process in children does not involve eyesight because of the insufficiently developed visual–motor coordination [12,35]. On the other hand, based on research involving adults, it has been suggested that humans’ automatic postural response systems are unaffected by the absence of vision from birth [5]. These observations caught our attention, and as a result, we decided to carry out this research.

The present study’s findings show no differences in balance parameters between the BL and NE groups in the trials on the foam surface, with eyes open or closed. It is likely that proprioception, the primary source of information for BL subjects, makes it possible for them to perform stabilometric tests as effectively as NE subjects. We are aware that the participants of our study represent a wide range of age groups, making it difficult to compare our results to other studies. Because of this, we discuss our findings separately by reference to studies involving only adults or children. As regards the former, Nakata and Yabe, in their study, compared the automatic postural responses in congenitally blind and sighted male subjects aged 19–24 years. During the trials, four types of perturbations (forward and backward translations and toe up and down rotations) were randomly applied to induce postural responses in the subjects. The authors reported no differences between the postural responses of blind and sighted subjects to platform displacements, and as a result, they postulated that the ability to control postural balance during perturbations of this type was not affected by vision loss from birth. Hence, it seems that the absence of vision from birth in humans does not affect the automatic postural response systems [36].

On the other hand, Rogge et al. carried out a study in which balance training was provided to blind adult individuals as well as age- and gender-matched sighted controls. Assessment of the effects showed similar balance performance improvement in both groups [37]. Furthermore, changes in the vestibular and proprioceptive areas responsible for self-motion processing were shown in the grey matter, which would support our speculations of a positive effect of training on foam mats.

Other researchers, however, have reported poorer functioning of the balance system in blind or partially sighted populations. Houwen et al. [15] conducted a study involving children (7–10 years of age) with visual impairment and normally sighted individuals. The authors reported poorer static and dynamic balance performance in the former group, with no significant differences between children with moderate and severe visual impairments. These findings are consistent with the observations made by Skaggs and Hopper [38]. They reviewed literature focusing on the psychomotor abilities of individuals with visual impairments and found that some studies reported poorer balance control in these individuals compared to normally sighted subjects. Research focusing on similar issues in a cohort of children (aged 10–16 years) with and without hearing impairments was conducted by Wierzbicka-Damska et al. [39]. Balance control was assessed using a stabilometric platform in trials with eyes closed and open. Mean values of the related parameters reflected the slightly better performance of the subjects with hearing impairment. The authors argue that this is linked with more effective use of proprioceptors and cutaneous receptors. Similar results were identified by Walicka-Cupryś et al. [30] in a large cohort of 228 children aged 8–16, including 65 deaf subjects. In these two studies, in tests with no visual input (trials with eyes closed), the findings showed better balance parameters in the study groups. It is possible that individuals with visual or hearing impairment more effectively focus on maintaining balance, which may explain why they achieve similar or better scores in postural control tasks compared to normally developed peers. We also assume that BL individuals may be more attentive to any changes in the floor surface to avoid obstacles or falling, which they learn while walking unassisted with a cane. The contribution of proprioceptive inputs may be relatively higher in their daily life.

The present study shows a relationship between the type of floor surface and balance control parameters in BL subjects compared to NE individuals. In the BL group, there were differences in the values measured during trials with eyes open on stable versus unstable surfaces. In the NE group, the results did not differ similarly: the differences were only observed in the mean amplitude and the sway area in trials with eyes open, with higher values measured on the foam surface compared to the solid surface. This observation gains significance in light of the evidence suggesting that up until 12 or even 15 years of age, children cannot fully use vestibular and visual stimuli in the process of balance control. According to Paszko-Patej et al. [14], blind or partially sighted subjects can be expected to be more focused on the tasks performed, which results in the ability to maintain a more stable posture. However, the present study found no such effect in the BL group; it was only observed that the parameters assumed lower values in trials which involved standing on a stable surface with eyes closed in the BL group compared to the NE group, but these differences were not statistically significant. These findings suggest that proprioceptive information was used to maintain balance. This stipulation is also supported by the age of the subjects and their inability to use visual and vestibular inputs in balance control [8,9,10,12,35] in trials with eyes closed. The differences in COP behaviour between the two groups may suggest the greater proprioceptive sensibility of the BL subjects.

In summary, it can be stipulated that proprioception plays an essential role in balance control, given the fact that higher balance parameters are observed in BL subjects on an unstable surface. This information may be helpful for therapists working with BL individuals. It should be taken into account by those designing therapeutic programs for this population. Foam mats and other unstable surfaces should be used in training pro-grammes. In everyday life, blind people should experience as many exteroceptive stimuli, different textures, and unstable surfaces as possible. Consequently, further research is needed to investigate this issue in more detail to facilitate a more accurate assessment of postural stability in BL individuals.

### Limitations

The presented study has some limitations. The most significant is the wide age deviation of the respondents. We surveyed all willing persons who applied from the Polish Association of the Blind, Podkarpackie Branch. To partially offset this deviation, we matched the group 1:1, considering the age and gender. In addition, to ensure homogeneity of the study group, only people blind from birth qualified; all participants were physically inactive. We also recognise that balance and proprioception can vary significantly with age. Therefore, further research is needed that includes the division into age groups: children, adolescents, adults, and older people with BL. We think it would be interesting to compare balance in these age groups since involutionary changes occur in all systems of the human body, including those responsible for balance. A lack of clear-cut differences between the groups may be associated with the subject’s age and the related balance system immaturity and lability, characteristic of children and adolescents up to 15–16 years of age. This may also be associated with the less important roles of the vestibular and visual systems in balance control.

On the other hand, the data suggest that proprioception is the primary source of afferent input in balance control in BL subjects. As mentioned above, the group studied is individuals who have been blind since birth, that is, those who do not respond to all visual stimuli. It would also be worthwhile to conduct further studies on balance assessment according to the severity of the visual impairment and consider the impairment of monocular and binocular vision. In our study, all the participants were physically inactive, so it is necessary to continue the study with a consideration of physically active people with BL, taking into account the level or type of physical activity practised.

## 5. Conclusions

The present study’s findings did not show poorer balance in the BL group compared to the NE group. However, in the tests on the unstable surface, considerable sensitivity to the effects of the interfering factor was observed in the BL group. Proprioception plays an important role in balance control, given that a change in the surface leads to increased balance parameters in subjects with visual dysfunction. This explicitly shows it is necessary to develop body balance skills in blind subjects, by improving their proprioceptive sensibility and by building their spatial awareness of the body location. Foam mats and other unstable surfaces should be used in the training programme. In everyday life, blind people should experience as many exteroceptive stimuli, different textures, and unstable surfaces as possible.

## Figures and Tables

**Table 1:** Comparison of the results on the platform, foam surface versus solid surface, with eyes open and eyes closed, in the group of blind or partially sighted subjects.

**Parameters** **Eyes Open**	**BL Foam Surface n = 40**			**BL Solid Surface n = 40**			**Significance (*p*)**	
	$\bar{x}$	**Me**	**s**	$\bar{x}$	**Me**	**s**	**Z**	***p***
sway path	299.35	323.5	79.96	265.9	262.5	82.95	2.62	0.009 **
mean amplitude	3.97	3.55	1.63	3.45	3.3	1.76	1.53	0.126
mean velocity	9.94	10.25	2.62	8.93	8.75	2.76	2.6	0.009 **
sway area	383.8	413.5	225.62	293.85	272.5	227.29	2.39	0.017 *
LWAP-EO *	16.7	15.5	6.17	11.25	8.0	7.31	2.88	0.004 **
LWML-EO **	14.25	13.5	9.3	17.35	16.5	9.64	1.31	0.191
**Parameters** **Eyes Closed**	**BL Foam Surface n = 40**			**BL Solid Surface n = 40**			**Significance (*p*)**	
	$\bar{x}$	**Me**	**s**	$\bar{x}$	**Me**	**s**	**Z**	***p***
sway path	331.05	336.5	104.45	265.55	248.0	95.62	2.76	0.006 **
mean amplitude	3.78	3.9	1.03	2.94	2.55	1.77	2.17	0.030 *
mean velocity	10.94	11.2	3.49	8.87	8.25	3.12	2.78	0.006 **
sway area	404.15	401.5	237.9	234.05	149.0	270.78	2.58	0.009 **
LWAP-EC *	17.2	17.0	4.31	17.2	18.5	7.3	0.34	0.737
LWML-EC **	24.0	25.0	12.56	21.3	21.0	7.68	0.99	0.323

* LWAP—number of COP deflections along the sagittal plane; ** LWML—number of COP deflections along the frontal plane; EO—eyes open; EC—eyes closed; x—sample mean; Me—median; s—sample standard deviation; Z—Wilcoxon matched-pairs test.

**Table 2:** Comparison of the results on the platform, foam surface versus solid surface, with eyes open and eyes closed, in the control group.

**Parameters** **Eyes Open**	**NE Foam Surface n = 40**			**NE Solid Surface n = 40**			**Significance (*p*)**	
	$\bar{x}$	**Me**	**s**	$\bar{x}$	**Me**	**s**	**Z**	***p***
sway path	292.6	284.0	79.82	260.3	235.0	80.15	1.53	0.126
mean amplitude	3.54	3.5	1.35	2.63	2.2	1.48	2.13	0.033 *
mean velocity	9.75	9.45	2.66	8.68	7.8	2.68	1.53	0.126
sway area	351.45	305.5	206.24	208.95	187.0	135.73	2.17	0.030 *
LWAP-EO *	14.15	13,0	6.69	14.95	15.5	9.44	0.08	0.936
LWML-EO **	19.0	17.0	10.16	24.5	23.0	13.25	1.62	0.104
**Parameters** **Eyes Closed**	**NE Foam Surface n = 40**			**NE Solid Surface n = 40**			**Significance (*p*)**	
	$\bar{x}$	**Me**	**s**	$\bar{x}$	**Me**	**s**	**Z**	***p***
sway path	327.8	340.0	89.6	312.5	331.0	106.0	0.41	0.681
mean amplitude	4.6	3.9	3.3	3.1	2.6	1.9	1.94	0.052
mean velocity	10.9	11.3	3.0	10.4	11.1	3.5	0.39	0.695
sway area	433.9	400.5	294.0	279.4	254.0	187.4	1.94	0.052
LWAP-EC *	16.0	12.5	9.6	19.8	17.5	9.0	1.83	0.067
LWML-EC **	26.5	23.0	14.5	33.5	27.5	19.2	1.21	0.227

* LWAP—number of COP deflections along the sagittal plane; ** LWML—number of COP deflections along the frontal plane; EO—eyes open; EC—eyes closed; x—sample mean; Me—median; s—sample standard deviation; Z—Wilcoxon matched-pairs test.

**Table 3:** Comparison of the results on the platform between the groups; foam surface, eyes open and eyes closed.

**Parameters** **Eyes Open**	**BL Foam Surface n = 40**			**NE Foam Surface n = 40**			**Significance (*p*)**	
	$\bar{x}$	**Me**	**s**	$\bar{x}$	**Me**	**s**	**U**	***p***
sway path	299.4	323.5	80.0	292.6	284.0	79.8	0.43	0.665
mean amplitude	4.0	3.6	1.6	3.5	3.5	1.4	0.84	0.401
mean velocity	9.9	10.3	2.6	9.8	9.5	2.7	0.38	0.705
sway area	383.8	413.5	225.6	351.5	305.5	206.2	0.55	0.579
LWAP-EO *	16.7	15.5	6.2	14.2	13.0	6.7	1.36	0.175
LWML-EO **	14.3	13.5	9.3	19.0	17.0	10.2	−1.42	0.155
**Parameters** **Eyes Closed**	**BL Foam Surface n = 40**			**NE Foam Surface n = 40**			**Significance (*p*)**	
	$\bar{x}$	**Me**	**s**	$\bar{x}$	**Me**	**s**	**U**	***p***
sway path	331.1	336.5	104.5	327.8	340.0	89.6	0.15	0.882
mean amplitude	3.8	3.9	1.0	4.6	3.9	3.3	−0.23	0.818
mean velocity	10.9	11.2	3.5	10.9	11.3	3.0	0.01	0.989
sway area	404.2	401.5	237.9	433.9	400.5	294.0	0	1.000
LWAP-EC *	17.2	17.0	4.3	16.0	12.5	9.6	1.26	0.207
LWML-EC **	24.0	25.0	12.6	26.5	23.0	14.5	−0.2	0.839

* LWAP—number of COP deflections along the sagittal plane; ** LWML—number of COP deflections along the frontal plane; EO—eyes open; EC—eyes closed; x—sample mean; Me—median; s—sample standard deviation; U—Mann–Whitney U test.

**Table 4:** Comparison of the results on the platform between the groups; solid surface, eyes open, eyes closed.

**Parameters** **Eyes Open**	**BL Solid Surface n = 40**			**NE Solid Surface n = 40**			**Significance (*p*)**	
	$\bar{x}$	**Me**	**s**	$\bar{x}$	**Me**	**s**	**U**	***p***
sway path	265.90	262.50	82.95	260.30	235.00	80.15	0.28	0.776
mean amplitude	3.45	3.30	1.76	2.63	2.20	1.48	2.37	0.018 *
mean velocity	8.93	8.75	2.76	8.68	7.80	2.68	0.24	0.808
sway area	293.85	272.50	227.29	208.95	187.00	135.73	1.60	0.111
LWAP-EO *	11.25	8.00	7.31	14.95	15.50	9.44	−1.11	0.267
LWML-EO **	17.35	16.50	9.64	24.50	23.00	13.25	−1.64	0.102
**Parameters** **eyes closed**	**BL solid surface n = 40**			**NE solid surface n = 40**			**Significance (*p*)**	
	$\bar{x}$	**Me**	**s**	$\bar{x}$	**Me**	**s**	**U**	***p***
sway path	265.55	248.00	95.62	312.45	331.00	105.96	−1.38	0.168
mean amplitude	2.94	2.55	1.77	3.05	2.55	1.85	−0.28	0.776
mean velocity	8.87	8.25	3.12	10.41	11.05	3.53	−1.37	0.172
sway area	234.05	149.00	270.78	279.40	254.00	187.40	−1.26	0.208
LWAP-EC *	17.20	18.50	7.30	19.80	17.50	8.99	−0.74	0.456
LWML-EC **	21.30	21.00	7.68	33.50	27.50	19.17	−1.84	0.065

* LWAP—number of COP deflections along the sagittal plane; ** LWML—number of COP deflections along the frontal plane; EO—eyes open; EC—eyes closed; x—sample mean; Me—median; s—sample standard deviation; U—Mann–Whitney U test.

## Data Availability

Not applicable.
